# A newly designed duodenal stent enables stent placement for jejunal obstruction

**DOI:** 10.1055/a-2717-1417

**Published:** 2025-10-21

**Authors:** Shota Harai, Susumu Hijioka, Yoshikuni Nagashio, Daiki Yamashige, Yutaka Saito, Takuji Okusaka

**Affiliations:** 1 68380 Department of Hepatobiliary and Pancreatic Oncology, National Cancer Center Hospital, Tokyo, Japan; 2 Endoscopy Division, National Cancer Center Hospital, Tokyo, Japan

An 80-year-old woman who had undergone surgery for pancreatic tail cancer received chemotherapy for peritoneal dissemination recurrence, but the response was poor. She developed vomiting due to stenosis in the ascending part of the duodenum caused by tumor progression ( Fig. 1 **a** ). After decompression with an ileus tube, a duodenal stent was placed ( Fig. 1 **b, c** ), resulting in temporary relief. However, vomiting recurred after two weeks, and computed tomography (CT) revealed a jejunal stenosis approximately 20 cm distal to the ligament of Treitz ( Fig. 2 **a, b** ). An ileus tube was reinserted.

**Fig. 1:**
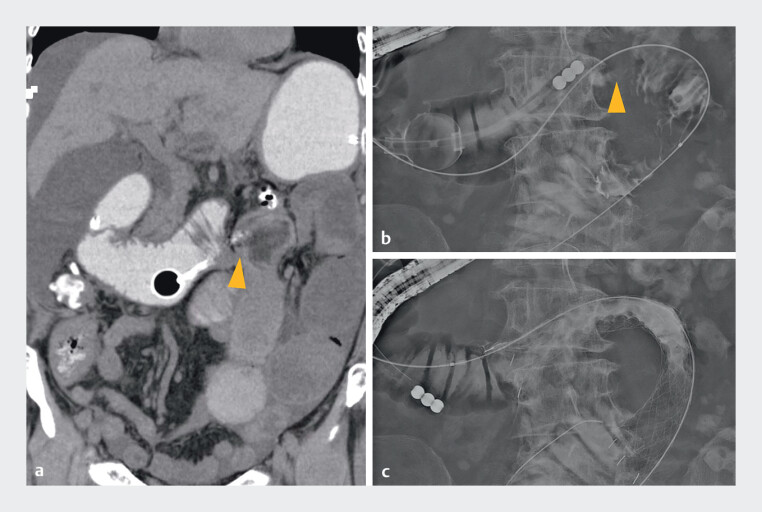
Duodenal stent placement for stenosis in the transverse part of the duodenum. **a** On the coronal view of the computed tomography (CT), a stricture in the transverse part of the duodenum (arrowhead) and dilatation of the proximal bowel were observed. **b** The endoscopic duodenal contrast study confirmed stenosis (arrowhead) in the transverse part of the duodenum. **c** A stent was placed at the stenosis site.

**Fig. 2:**
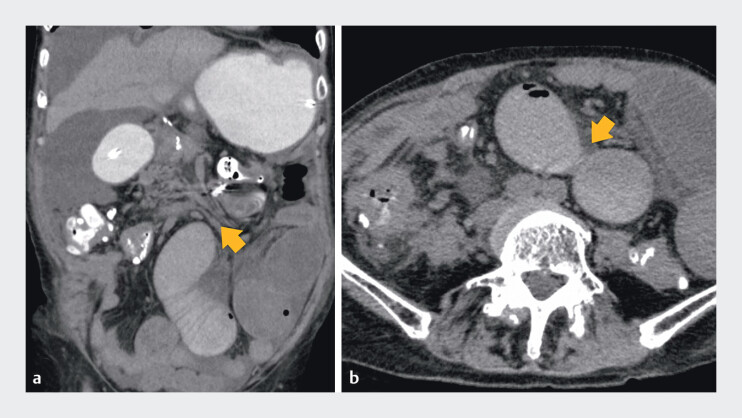
CT image showing a malignant stricture in the jejunum. **a** The stenosis was located approximately 20 cm from the Treitz ligament to the anal side (arrow). **b** Axial view.

Given that the narrowing was confined to a localized segment of the proximal jejunum, stent placement was considered appropriate. A colonoscope (CF-H260; Olympus, Tokyo, Japan) was used. A catheter and a 0.025-inch guidewire (GW) were inserted through the existing stent ( Video 1 ). Contrast imaging showed a stenosis 3 cm in length consistent with CT findings ( Fig. 3 **a** ). A 0.035-inch GW was added to enhance stent deliverability. Two duodenal stents (22 mm × 12 cm and 22 mm × 8 cm; JENTLLY NEO Duodenal Stent; Japan Lifeline, Tokyo, Japan) were selected to prevent kinking ( Fig. 3 **b** ). Both showed good trackability. The stents were accurately placed, and contrast confirmed adequate distal flow ( Fig. 3 **c** ). Postoperatively, ileus tube drainage decreased, and CT showed improvement in dilatation ( Fig. 4 **a–c** ). The patient resumed oral intake.

**Fig. 3:**
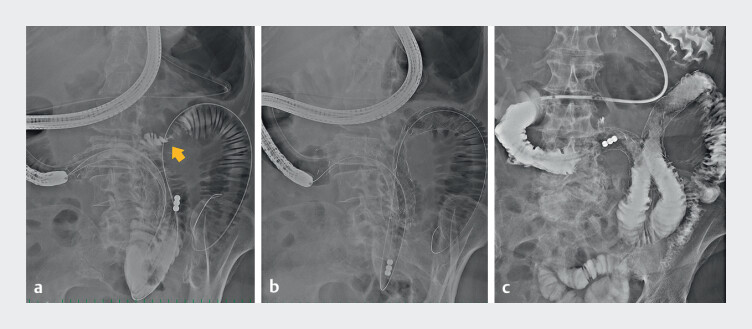
Stent placement for a malignant jejunal stricture. **a** The endoscopic duodenal contrast study confirmed stenosis (arrow) in the jejunum. **b** A stent (22 mm × 12 cm) was placed with sufficient length to prevent kinking on the anal side. An additional stent (22 mm × 8 cm) was placed on the oral side. **c** Contrast from the ileus tube confirmed smooth flow through the stent lumen into the distal bowel.

**Fig. 4:**
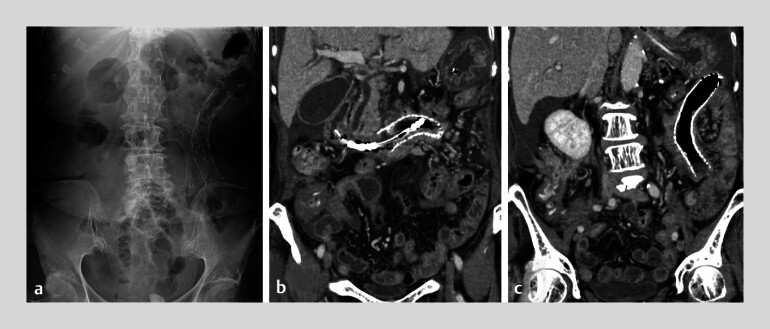
Imaging after duodenal stenting. **a** X-ray after duodenal stenting. **b** Duodenal stent at the ascending part of the duodenum. **c** Duodenal stent at the jejunum.

Duodenal stent placement for jejunal obstruction.Video 1

Stent placement is a minimally invasive alternative to surgery for gastrointestinal strictures 1 2 3 . However, placement in distal jejunal strictures is technically difficult due to the need to traverse the ligament of Treitz and navigate tortuous anatomy 4 . In this case, the newly designed stents’ strong outer sheath and low-friction inner surface enabled smooth advancement and accurate deployment in a deep, angulated jejunal segment. This report demonstrates a feasible stenting approach for malignant jejunum obstruction and may represent a valuable therapeutic option.

Endoscopy_UCTN_Code_TTT_1AO_2AZ

## Notes

Endoscopy E-Videos https://eref.thieme.de/e-videos
*E-Videos* is an open access online section of the journal *Endoscopy* , reporting on interesting cases and new techniques in gastroenterological endoscopy. All papers include a high-quality video and are published with a Creative Commons CC-BY license. Endoscopy E-Videos qualify for HINARI discounts and waivers and eligibility is automatically checked during the submission process. We grant 100% waivers to articles whose corresponding authors are based in Group A countries and 50% waivers to those who are based in Group B countries as classified by Research4Life (see: https://www.research4life.org/access/eligibility/ ). This section has its own submission website at https://mc.manuscriptcentral.com/e-videos .
